# Predicting risk of severe neonatal outcomes in preterm infants born from mother with prelabor rupture of membranes

**DOI:** 10.1186/s12884-022-04855-0

**Published:** 2022-07-04

**Authors:** Lu Zhuang, Zhan-Kui Li, Yuan-Fang Zhu, Rong Ju, Shao-Dong Hua, Chun-Zhi Yu, Xing Li, Yan-Ping Zhang, Lei Li, Yan Yu, Wen Zeng, Jie Cui, Xin-Yu Chen, Jing-Ya Peng, Ting Li, Zhi-Chun Feng

**Affiliations:** 1 Senior Department of Pediatrics, the Seventh Medical Center of PLA General Hospital, Beijing, China; 2 National Engineering Laboratory for Birth Defects Prevention and Control of Key Technology, Beijing, China; 3 Beijing Key Laboratory of Pediatric Organ Failure, Beijing, China; 4 Northwest Women’s and Children’s Hospital, Xi’an, Shanxi province China; 5 Shenzhen Baoan Women’s and Children’s Hospital, Jinan University, Shenzhen, Guangdong province China; 6 School of Medicine, Chengdu Women’s and Children’s Central Hospital, University of Electronic Science and Technology of China, Chengdu, China

**Keywords:** Prognostic nomogram, Severe neonatal outcomes, Preterm prelabor rupture of membranes

## Abstract

**Background:**

Perinatal complications are common burdens for neonates born from mother with pPROM. Physicians and parents sometimes need to make critical decisions about neonatal care with short- and long-term implications on infant’s health and families and it is important to predict severe neonatal outcomes with high accuracy.

**Methods:**

The study was based on our prospective study on 1001 preterm infants born from mother with pPROM from August 1, 2017, to March 31, 2018 in three hospitals in China. Multivariable logistic regression analysis was applied to build a predicting model incorporating obstetric and neonatal characteristics available within the first day of NICU admission. We used enhanced bootstrap resampling for internal validation.

**Results:**

One thousand one-hundred pregnancies with PROM at preterm with a single fetus were included in our study. SNO was diagnosed in 180 (17.98%) neonates. On multivariate analysis of the primary cohort, independent factors for SNO were respiratory support on the first day,, surfactant on day 1, and birth weight, which were selected into the nomogram. The model displayed good discrimination with a C-index of 0.838 (95%CI, 0.802–0.874) and good calibration performance. High C-index value of 0.835 could still be reached in the internal validation and the calibration curve showed good agreement. Decision curve analysis showed if the threshold is > 15%, using our model would achieve higher net benefit than model with birthweight as the only one predictor.

**Conclusion:**

Variables available on the first day in NICU including respiratory support on the first day, the use of surfactant on the first day and birthweight could be used to predict the risk of SNO in infants born from mother with pPROM with good discrimination and calibration performance.

## Introduction

Preterm prelabor rupture of membranes (pPROM) complicates 2.5 ~ 3% of pregnancies and is responsible for one third of preterm birth [1, 2]. The practice bulletins released by the American Congress of Obstetricians and Gynecologists (ACOG) and the Royal College of Obstetricians and Gynaecologists (RCOG) endorses antibiotics, corticosteroid, induction or expectant management [1, 3].

Perinatal complications are common burdens for infants born from mother with pPROM. The consequences of pPROM for neonates are premature birth complications [4], short-term neonatal disease(neonatal pneumonia, neonatal sepsis, et al.) [5] and long-term disability (blindness, deafness and cerebral palsy) [6]. It is reported that the risk of neurodevelopmental impairment for neonates would be higher if the mother who suffer from preterm PROM with intrauterine inflammation [7, 8], and the risk of neonatal white matter damage would be associated with early gestational age at membrane rupture [9]. The most common reported complication of prematurity is respirator [4]. Necrotizing enterocolitis (NEC) and intraventricular hemorrhage (IVH), and sepsis are also reported to be associated with preterm birth.

Perinatal mortality and the incidence of severe neonatal morbidity are higher in those preterm infants with lower birthweight [4, 10, 11]. There were several studies developed graphical tools or model to predict survival or survival without severe morbidities in preterm infants [12–14]. To facilitate prediction in PROM pregnancies, Jose R. Ducan et al. used clinical variables obtainable before delivery for severe neonatal outcomes and found estimated fetal weight showed significant effect on the prediction probability of the SNO [15]. Physicians and parents sometimes need to make critical decisions about neonatal care with short- and long-term implications on infant’s health and families. As postnatal clinical data could be available to neonatologists, here we conducted a study to find the association of clinical variables obtained before or after delivery for severe neonatal outcomes (SNO) and develop a valid but simple clinical tool using these variables to assess the risk of these outcomes.

## Materials and methods

This was a further study of the previous cohort (MCPPNC, Multi-center Cohort of Pregnancies with PROM and their Neonates in China), a prospective, multi-center cohort study aimed to describe the epidemiology of PROM and assess the influence of the implementation of the guideline [2].

As described in our previously published study [2], PROM was defined as rupture of membranes before the on-set of labor [1]. PROM was confirmed by pooling and positive PROM test (PH test or insulin-like growth factor binding protein 1 detection test). Briefly, PROM pregnancies were recruited between August 1, 2017, to March 31, 2018 from three hospitals (Shenzhen Baoan Maternity and Children’s Hospital, Xibei Women and Children’s Hospital and Chengdu Women and Children’s Hospital) in China. Participants whose estimated gestational age (GA) of ≥ 42 weeks and < 24 weeks were excluded. Demographic and clinical data were collected. This study was approved by the Ethical Committee of PLA Army General Hospital, China (2017–42) and assigned on the Protocol Registration and Results System of ClinicalTrials.gov (NCT03251898).

In the present study, we included pregnancies with PROM at preterm (estimated GA < 37 weeks from MCPPNC) with a single fetus. The outcome of neonates who were hospitalized in neonatology department were followed until they were leave hospital.

Severe neonatal outcomes (SNO) were defined as the following: necrotizing enterocolitis (NEC), respiratory distress syndrome (RDS), intraventricular hemorrhage (IVH), neonatal sepsis, bronchopulmonary dysplasia (BPD) and neonatal death. RDS was defined as surfactant deficiency based on clinical or radiologic evidences [16]. According to Bell’s staging, stage II and III was defined as NEC [17], IVH was defined according to the Papille classification [18]. Sepsis was defined by the presence of clinical symptoms and a positive culture from blood or cerebrospinal fluid samples. BPD was diagnosed according to NIH 2018 definition [19].

As defined in our previous published study, clinical chorioamnionitis was characterized by maternal fever, leukocytosis, maternal and/or foetal tachycardia and uterine tenderness [2, 20]. Subclinical/histologic chorioamnionitis which is asymptomatic, was confirmed by pathological section of placenta. We defined degree I, II and III meconium-stained amniotic fluid as “amniotic fluid pollution” [2, 21, 22]. Gestational hypertensive (GH) is defined as a systolic blood pressure of 140 mm Hg or more or a diastolic blood pressure of 90 mm Hg or more, or both, on two occasions at least 4 h apart after 20 weeks of gestation in a woman with a previously normal blood pressure [2, 23]; The definition of diabetes mellitus arising in pregnancy (DMP) were according to the international classification of Diseases (ICD), 11^th^ Revision (https://icd.who.int/browse11/l-m/en#/http://id.who.int/icd/entity/1320503631) [2];

### Statistical analyses

Statistical analysis was carried out with the R software (Version 4.1.2; https://www.R-project.org). rms (version 6.0–1) [24] for logistic regression modelling, pROC (version 1.16.2) [25] for C-statistic calculations, rmda (version 1.6) [26] for decision curve analysis.

Multivariable logistic regression analysis was performed to establish a predicting model. The clinical variables included in the regression model were “Respiratory support on the first day: Oxygen therapy (oxygen inhalation in incubator, oxygen inhalation with facemask, oxygen inhalation in oxygen chamber), Normal frequency ventilation (including the use of continuous positive airway pressure (CPAP) and synchronized intermittent mandatory ventilation (SIMV)), and High-frequency ventilation (HFO), the use of surfactant on the first day, clinical or subclinical chorioamnionitis, DMP, GH, birthweight, the use of antenatal corticosteroids, latency days from PROM to delivery, and amniotic fluid pollution”. The variables were selected by stepwise selection. The statistical significance levels were all two sided. All suspected predictors were incorperated to develop a prediction model for SNO risk by using the cohort.

The SNO nomogram was assessed by calibration curves. The model would not be considered to calibrate perfectly if there was a significant test statistic. The discrimination performance of the SNO nomogram was quantified by the Harrell’s C-index. The nonadherence nomogram was subjected to enhanced bootstrapping validation (1,000 bootstrap resamples) to calculate a relatively corrected C-index [27]. The clinical usefulness of the nomogram was determined by conducting decision curve analysis [28] and by quantifying the net benefits at different threshold probabilities for model development by simple predictor birth weight and complex predictors selected by stepwise method.

## Results

### Patients’ characteristics

**Fig. 1:**
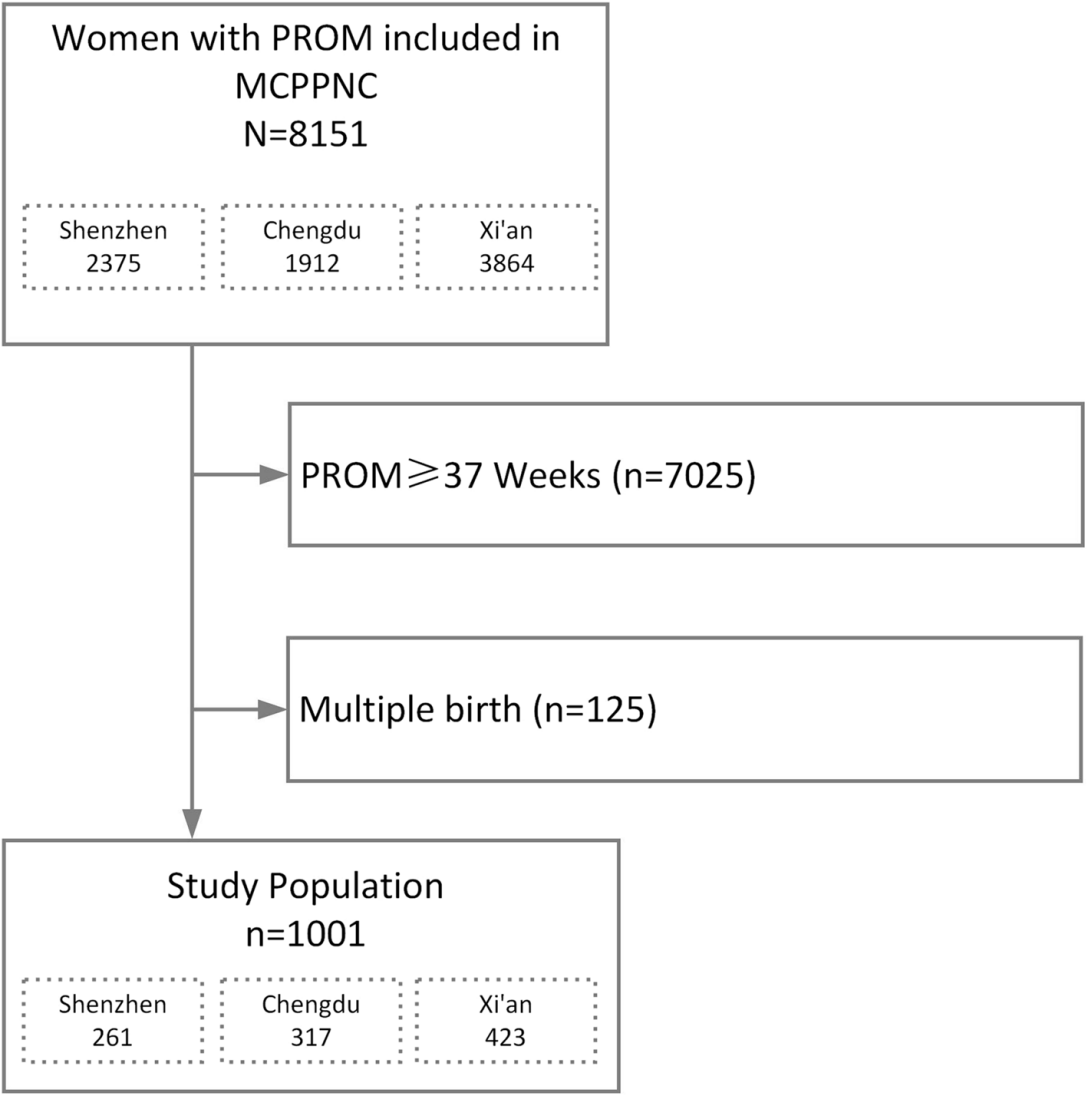
The flow chart of selection of study subjects from the 2017–2018 MCPPNC database

From August 1, 2017, to March 31, 2018, a total of 8151 women were treated for PPROM in the three hospitals during the study period. Of these, 1001 women met inclusion criteria and were included in analysis (Fig. 1). Demographic and candidate predictors and their proportions of missingness are shown in Table 1. SNO was diagnosed in 180(17.98%) neonates. RDS and intracranial hemorrhage were the most common SNO and were diagnosed in 84 neonates respectively. NEC was diagnosed in 14 newborns (1.40%). Sepsis was diagnosed in 27(2.70%) neonates. BPD was diagnosed in 7(0.70%) neonates. Multiple SNO was present in 41 neonates.

**Table 1:** Baseline characteristics of the study cohort, stratified by severe neonatal outcomes

	**Severe neonatal outcomes**	
	**No**	**Yes**
**Race (0 missing)**		
Yellow	821	180
** Mother’s age, years, mean (SD) (1 missing)**	30.68 ± 4.58	30.57 ± 4.50
** Latency, days (0 missing)**	1.95 ± 3.08	3.46 ± 4.22
** GA at PPROM, weeks, mean (SD) (0 missing)**	34.98 ± 2.09	31.83 ± 2.93
** GA at delivery, weeks, mean (SD) (1 missing)**	35.23 ± 1.93	32.29 ± 2.74
**Nulliparity (0 missing)**		
No	353	88
Yes	468	92
**Gestational diabetes (0 missing)**		
No	661	147
Yes	160	33
**Hypertensive disorders of pregnancy (0 missing)**		
No	799	176
Yes	22	4
**Clinical or subclinical chorioamniotis (0 missing)**		
Yes	209	80
No	612	100
**Amniotic fluid pollution (31 missing)**		
Yes	31	10
No	773	159
**Use of antenatal corticosteroids (0 missing)**		
Yes	321	97
No	500	83
**Neonates’ Gender (0 missing)**		
Female	353	79
Male	468	101
** Neonates’ Birthweight (0 missing)**	2528.4 ± 515.9	1888.7 ± 577.8
**Use of surfactant on the 1st day (0 missing)**		
Yes	2	49
No	819	131
**Respiratory support**		
None	766	86
Oxygen therapy	10	4
Normal frequency ventilation	41	69
High-frequency ventilation	4	21

### Predictors selection and prediction model development

**Fig. 2:**
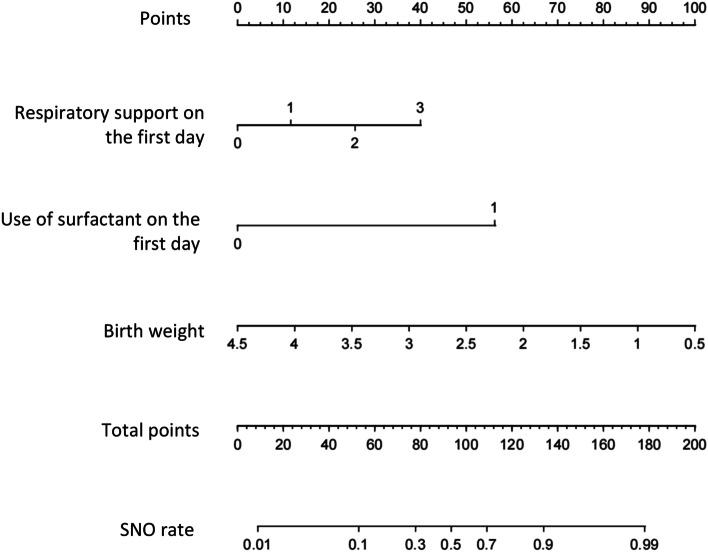
Developed SNO nomogram. The SNO nomogram was developed in the cohort, with Respiratory support on the first day, the use of surfactant on the first day and birthweight incorporated. The level of Respiratory support: 0, No respiratory support; 1 = Oxygen therapy (oxygen inhalation in incubator, oxygen inhalation with facemask, oxygen inhalation in oxygen chamber); 2, Normal frequency ventilation (including the use of continuous positive airway pressure (CPAP) and synchronized intermittent mandatory ventilation (SIMV)); 3 = High-frequency ventilation (HFO)

In stepwise selection, 3 predictors were retained on the basis of 1001 neonates. These predictors were Respiratory support on day 1, surfactant on day 1 and birth weight. Logistic regression was conducted based on the above three predictors. The odds ratios and 95% CIs for the 3 factors were presented in Table 2. The model was developed and presented as the nomogram (Fig. 2).

**Table 2:** Odds Ratio Estimates and 95% CIs for Factors Selected in the Prediction Model Developed

Variables	*β*	Wald	OR[95%CI]	*p*
**Respiratory support**				
Oxygen therapy	0.632	1.014	1.881[0.550, 6.437]	0.314
Normal frequency ventilation	1.400	25.322	4.040[2.345, 6.958]	< 0.001
High-frequency ventilation	2.173	7.236	8.781[1.803, 42.770]	0.007
**Surfactant on Day 1**	3.055	15.603	21.221[4.661, 96.622]	< 0.001
**Birth weight**	-1.358	49.296	0.257 [0.176, 0.376]	< 0.001

### Apparent performance of the nonadherence risk nomogram in the cohort

**Fig. 3:**
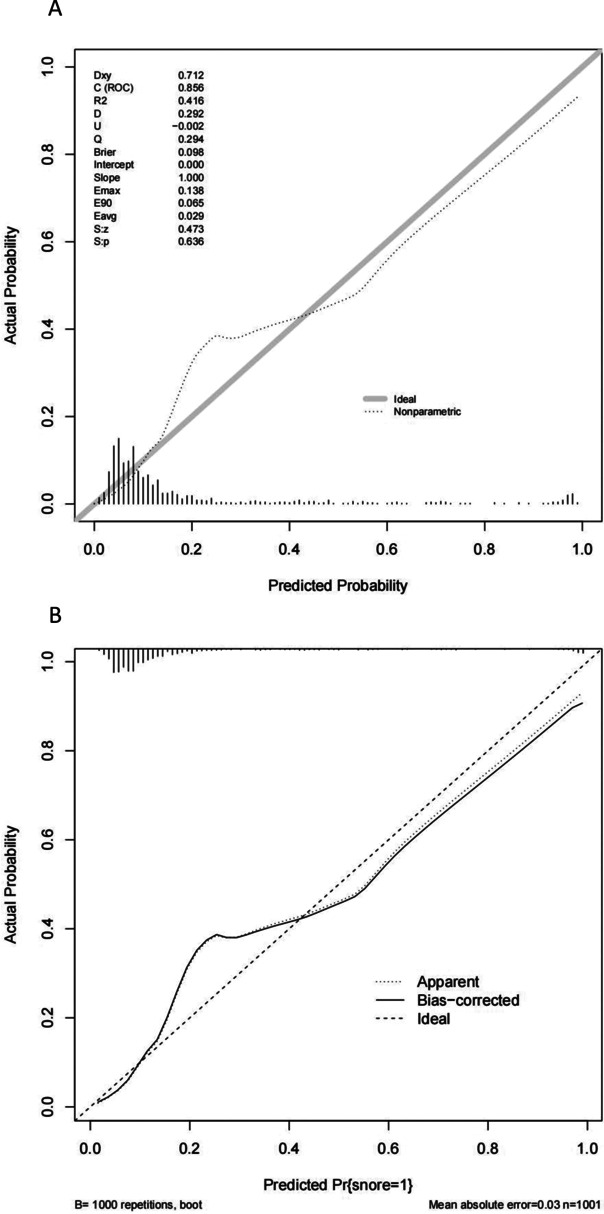
Calibration curves. **A**. Calibration curves of the SNO nomogram. **B**. Calibration curves of the internal validation. The x-axis represents the predicted SNO risk. The y-axis represents the actual occurrence of SNO. The diagonal dotted line represents a perfect prediction by an ideal model. The solid line represents the performance of the nomogram, of which a closer fit to the diagonal dotted line represents a better prediction

The calibration curve of the nomogram for the prediction of SNO risk in preterm neonates demonstrated good agreement in this cohort (Fig. 3A). The C-index for the prediction nomogram was 0.856 (95%CI: 0.824–0.889). Internal validation of the model using enhanced bootstrap method showed good reproducibility with C-index to be 0.852 which suggested the model’s good discrimination. Calibration curve of internal validation also shows good agreement (Fig. 3B).

### Clinical use

**Fig. 4:**
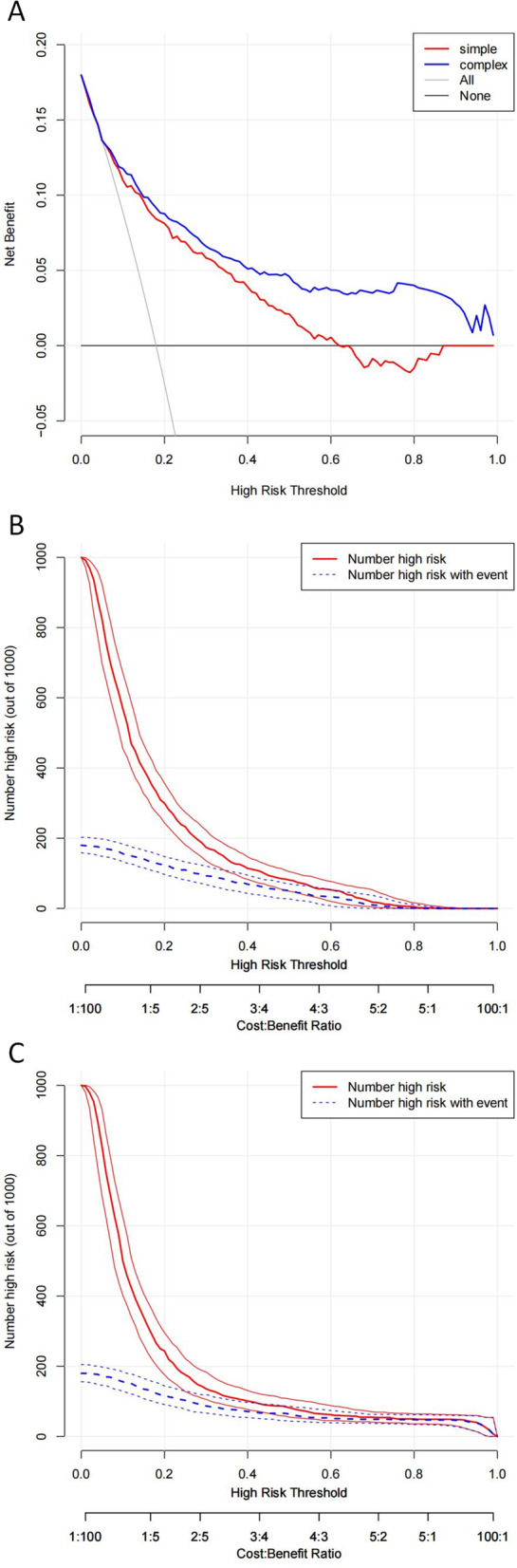
Decision curve analysis for the SNO nomogram. **A** Net benefit curves for the SNO nomogram. The y-axis measures the net benefit. The thin solid line represents the assumption that all patients are SNO. The thick solid line represents the assumption that no patients are SNO. The red line represents the SNO risk simple nomogram developed by only one variation “birth weight”. The blue line represents the SNO complex risk nomogram developed by variations “Respiratory support on the first day, the use of surfactant on the first day and birthweight”. **B** Clinical impact curves for the simple model. Clinical impact curve for the risk model base on variables including only birthweight. Of 1,000 patients, the heavy red solid line shows the total number who would be deemed high risk for each risk threshold. The blue dashed line shows how many of those would be true positives (SNO cases). **C** Clinical impact curves for the SNO complex model. Clinical impact curve for the risk model base on variables including Respiratory support, the use of surfactant on the first day and birthweight. Of 1,000 patients, the heavy red solid line shows the total number who would be deemed high risk for each risk threshold. The blue dashed line shows how many of those would be true positives (SNO cases)

The decision curve (Fig. 4A) shows that if the threshold is > 15%, using our model which included Respiratory support on day 1, surfactant on day 1 and birth weight to birth as predictors would achieve higher net benefit than the simple model with birthweight as the only one predictor. Clinical impact curve for the single model (use BWT as predictor) and the complex model (use Respiratory support on day 1, surfactant on day 1 and birth weight to birth as predictors) show the estimated number who would be declared high risk for each risk threshold and visually shows the proportion of those who are cases (true positives) (Figs. 4B and 4C).

## Discussion

We developed and validated a prognostic model for SNO among 1101 preterm neonates hospitalized in department of neonatology. The final model integrated three routinely available predictors and could be used at the point of admission for preterm neonates. Internal validation showed consistent discrimination and calibration in the cohort for development of the model. Our model provides a probability output that could indicate the chance of the individual under evaluation having the outcome. These predictions would enable clinicians to objectively assess deterioration risk to inform the need for interventions such as ongoing hospital admission, consideration for critical care, and initiation of therapeutic agents.

In the development of our model, factors that were supposed to affect neonatal outcomes were included. “Respiratory support on the first day, the use of surfactant on the first day and birthweight” were suggested to be the key individual factors that determine risk of SNO. These factors in our final model are also well-known predictors of survival in preterm infants [29, 30]. As expected, birthweight was the strongest predictor. Neonates who were received CPAP or SIMV were at risk (adjusted OR = 4.040, 95%CI [2.345, 6.958], *p* < 0.001) than those who did not need respiratory support. Those who received HFO were at high risk (adjusted OR = 8.781, 95%CI [1.803, 42.770], *p* = 0.007) of SNO. Neonates received surfactant on Day 1 showed great risk of SNO (adjusted OR = 21.221, 95%CI [4.661, 96.622], *p* < 0.001).

A recent study for prediction of SNO conducted by Jose R. Duncan et al. [15] reported that estimated fetal weight could be used as a clinical toll to calculate the prediction probability of SNO in PPROM. In their model, several variables available before delivery such as gestational age, diabetes mellitus, fetal growth restriction and the appearance of clinical chorioamnionitis et al. were enrolled in their model. However, only estimated fetal weight were left as the predictor. The findings in our study echoed the study that factors obtained before delivery including clinical or subclinical chorioamnionitis, DMP, GH, the use of antenatal corticosteroids, latency days from PROM to delivery and amniotic fluid pollution were all adjusted.

Another study in Canada [14] recruited over 6000 preterm infants born from 23 to 30 weeks of gestation to develop accurate predictors models for multiple severe perinatal outcomes. 8 predictors, including gestational age, small for gestational age, gender, inborn or outborn status, antenatal corticosteroid use, SNAPII score > 20, and receipt of surfactant and mechanical ventilation on the first day after NICU admission were enrolled. Although prenatal and postnatal indicators, such as first trimester ultrasound, last menstrual period (LMP) and neonatal data were used to determine gestational age for individual infant management in nowadays [31], the gestational age before birth in medical record was still mainly the one that estimated based on maternal recollection of the last normal menstrual period (LNMP) that would be fraught with error. Thus, we used birth weight instead of gestational age in our model.

Esteves JS et al. conducted a study included 61 pregnancies with PPROM from 18 to 26 weeks of gestation from Brazil. The investigators demonstrated the only predictor for survival was the birthweight with an AUC of 0.90 [32]. While our model which included “Respiratory support on the first day, the use of surfactant on the first day and birthweight” as predictors achieved higher net benefit than model with birthweight as the only one predictor if the threshold is > 15%. The clinical effect curve of the complex model also showed better result than that of the simple model.

The limitation of our study was that considering that model using a smaller training subset may exclude important risk factors that do not reach the required statistical significance threshold because of reduced power, we included all the data from 3 centers into our model for developing the prognostic nomogram. Therefore, we recommend that our model be validated in larger and more diverse populations. Second, there were lack of a more precise level of IVH.

## Conclusions

In conclusion, the birthweight, respiratory support on the first day, the receipt of surfactant, had association for SNO in neonates born from mother with PPROM. We presented a prediction nomogram that appears to accurately estimate the probability for severe neonatal outcomes in pregnancies complicated by PPROM. The prognostic nomogram need validation in more diverse and larger populations.

## Data Availability

The data that support the findings of this study are available from the Seventh Medical Centre, PLA general hospital but restrictions apply to the availability of these data, which were used under license for the current study, and so are not publicly available. Data are however available from the authors upon reasonable request and with permission of the Seventh Medical Centre, PLA general hospital.

## Abbreviations

ACOG: American Congress of Obstetricians and Gynecologists
CI: Confidence interval
CPAP: Continuous positive airway pressure
DMP: Diabetes mellitus arising in pregnancy
GA: Gestational age
GH: Gestational hypertensive
HFO: High-frequency ventilation
ICD: International classification of diseases
IVH: Intraventricular hemorrhage
MCPPNC: Multi-center Cohort of Pregnancies with PROM and their Neonates in China
NEC: Necrotizing enterocolitis
OR: Odds ratio
pPROM: Preterm prelabor rupture of membranes
RCOG: Royal College of Obstetricians and Gynaecologists
RDS: Respiratory distress
SIMV: Synchronized intermittent mandatory ventilation
SNO: Severe neonatal outcomes
